# The effects of pelvic belt use on pelvic alignment during and after pregnancy: a prospective longitudinal cohort study

**DOI:** 10.1186/s12884-019-2457-6

**Published:** 2019-08-22

**Authors:** Saori Morino, Mika Ishihara, Fumiko Umezaki, Hiroko Hatanaka, Mamoru Yamashita, Rika Kawabe, Tomoki Aoyama

**Affiliations:** 1 Department of Physical Therapy, Faculty of Comprehensive Rehabilitation, Osaka Prefecture University, 3-7-30 Habikino, Habikino-shi, Osaka, 583-8555 Japan; 2 Pilates Studio Wohl, Nagoya, Aichi Japan; 3 Kishokai Medical Corporation, Nagoya, Aichi Japan; 4 Department of Physical Therapy, Human Health Sciences, Graduate School of Medicine, Kyoto University, Kyoto, Japan

**Keywords:** Pregnancy, Pelvic belt, Pelvic alignment, Childbirth

## Abstract

**Background:**

Pelvic alignment changes during pregnancy and post-childbirth. Pelvic belts exert external forces that compress and stabilize the joints, and therefore, could influence pelvic alignment. However, limited information is available regarding this potential effect. Therefore, the purpose of this study is to investigate the influence of pelvic belt use on pelvic alignment during and after pregnancy.

**Methods:**

Data of 201 pregnant women in late pregnancy and 1 month after childbirth were used. Pelvic alignment measurements, including anterior and posterior pelvic width, pelvic asymmetry, and pelvic belt use during and after pregnancy were investigated. Participants were divided into four groups according to pelvic belt use: before and after childbirth (BAC), before childbirth only (BC), after childbirth only (AC), and non-use (NU). Then, an initial one-way ANOVA was conducted to compare the amount of change in pelvic alignment from late pregnancy to post-childbirth between the groups. After the initial analysis, a multivariate regression analysis was performed to determine the statistically significant differences between the groups to consider other factors that influenced pelvic alignment such as age, BMI, number of previous childbirths, vaginal delivery and pelvic asymmetry in late pregnancy. Next, a cutoff point for subgroup stratification based on the weekly duration of pelvic belt use and inter-group changes in pelvic alignment were compared.

**Results:**

As the result of the initial one-way ANOVA, the decrease in pelvic asymmetry from during pregnancy to postpartum for BAC was greater than that for AC. Moreover, multiple regression analysis showed that the effect of pelvic belt that was revealed in the initial analysis was statistical significance even after adjustment for other factors. Moreover, pelvic asymmetry in the BAC group decreased, compared to being increased or unchanged in the NU and AC groups when the group cutoff time was 7 h per week.

**Conclusions:**

Continuous and extended use of pelvic belts during and after pregnancy might be related to modifications of pelvic asymmetry in the perinatal period. Therefore, the instruction of correct and comfortable usage and the recommendation of continuous use of pelvic belt especially during pregnancy are required for prevention of some discomforts related to pelvic malalignment.

**Electronic supplementary material:**

The online version of this article (10.1186/s12884-019-2457-6) contains supplementary material, which is available to authorized users.

## Background

During and after pregnancy, joint laxity and swelling of the abdomen are associated with the fetal growth changes the pelvic alignment of women [1, 2]. For example, softening of the ligaments that join the pelvis occur because of pregnancy-related hormones such as relaxin which function to make space for fetal growth [3]. In addition, the pelvis widens as the abdomen enlarges with the advancing pregnancy and to facilitate the forthcoming labor and delivery [4]. Although widening of the pelvis is necessary for fetal growth and childbirth, significant problems such as separation of the pubic symphysis can occur when pelvic widening exceeds the limits of its range of motion and pelvic joint flexibility [5]. Pelvic asymmetry alters the body’s mechanics, placing strain on various body segments, which subsequently contributes to musculoskeletal pain [6, 7]. A difference in the amount of change on left and right sides of the body could cause maladaptive responses, such as altered movement patterns that can result in pain [8]. Since pelvic asymmetry is considered to be a cause of lumbopelvic pain among pregnant women [9], it can be said that this asymmetry causes definite discomfort. According to a study that investigated the differences in pelvic alignments among women, the anterior widths of the pelvises of pregnant and postpartum women were greater than those of never-pregnant women [10]. In other words, changes in pelvic alignment that occur in pregnancy might persist after pregnancy. As such, pelvic belts are popular among these women as a tool to prevent changes in pelvic alignment in addition to reduce lumbopelvic pain. The pelvic belt helps to stabilize the pelvis and is thought to be effective for relieving lumbar and pelvic pain [11]. It has been suggested that hip adduction forces in patients with pregnancy-related pelvic girdle pain is increased [12], the intensity of pelvic pain is reduced, and that daily activities are improved with pelvic belt use [13, 14]. These effects are thought to occur because the belts, which compress the pelvis externally, are thought to augment pelvic stability via additional closure forces in lumbopelvic disorders where stability is compromised [14, 15]. This function of the pelvic belt might affect the width of the pelvis [16]. Moreover, this pelvic compression function inhibits excessive movement of the pelvis joint and might also correct pelvic asymmetry.

There are few studies that have revealed the effects of pelvic belt use on pelvic alignment based on objective data and the effect especially in perinatal period was not revealed [17]. However, many women use pelvic belts even though the effects of the belt on pelvic alignment have not been confirmed scientifically. Moreover, a longitudinal survey investigating the influence of pelvic belt use during and after pregnancy has not been conducted, even though it is known that pelvic alignment changes as pregnancy progress. Therefore, this study was undertaken to longitudinally investigate the influence of pelvic belt use during pregnancy and after childbirth on pelvic alignment in the perinatal period.

### Definition of terms

Pelvic alignment: positional relation between the bones in the pelvis; During pregnancy: the duration when a woman has a baby growing inside her body; Late pregnancy: the time when second investigation was conducted in the current study (30 ± 0.60 weeks of pregnancy); After pregnancy, After childbirth: the period after giving birth; Perinatal period: the period both of during and after pregnancy; Investigation period: the timing when measurement of pelvic alignment was conducted in the current study.

## Methods

This is a part of a prospective longitudinal cohort study that investigated the association between pelvic alignment and lumbopelvic pain during pregnancy. In the current study, the association between the use of pelvic belts and pelvic alignment in perinatal period was investigated by using the information of pelvic alignment and pelvic belt use. The current study was conducted adhering to STROBE guidelines.

### Participants

**Fig. 1:**
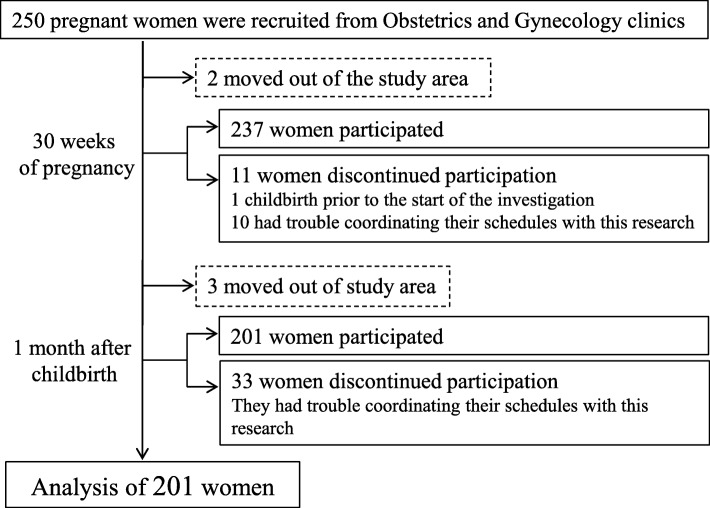
Flow diagram of the participants

Pregnant women were recruited from the Obstetrics and Gynecology clinics in Aichi Prefecture, Japan, between May and December 2014. The inclusion criteria: gestational age < 12 weeks and singleton pregnancy. Women with serious orthopedic disorders, neurological diseases, or high-risk pregnancies were excluded. Those who had already used pelvic belts as of the time of study recruitment were also excluded to remove any effects of pelvic belt use prior to pregnancy. Two hundred and fifty women who met the study inclusion criteria and agreed to participate were initially enrolled. Participants were observed during late pregnancy (30 ± 0.60 weeks of pregnancy) and after childbirth (31.5 ± 5.5 days after childbirth) in regular prenatal clinic visits. Among the initially enrolled participants, 13 and 36 women discontinued their participation during late pregnancy and after childbirth, respectively, for various reasons including hospital transfers, births that occurred before the late pregnancy investigation period, or personal feelings. Therefore, the data of 201 women were ultimately used in the analyses (Fig. 1).

### Questionnaires

Personal characteristics (age, height, weight at the time of recruitment, pre-pregnancy weight, and number of previous deliveries) were recorded at the time of recruitment. In addition, weights were recorded at each investigation period and birth weight was recorded after child birth. The weight, number of deliveries and birth weight as well as age were investigated because they are thought to be related to pelvic alignment. Pelvic belt use from 24 weeks of pregnancy, and that after childbirth were reviewed during the late pregnancy and post-childbirth investigation periods, respectively. The patients were queried as to whether or not they used pelvic belts, as well as how many days per week and the times in each day that the belts were used. If the participants were unclear as to any parts of the questionnaire, the measurers (midwives or physiotherapists) were available to answer their questions.

### Pelvic alignment

#### Measurement settings

**Fig. 2:**
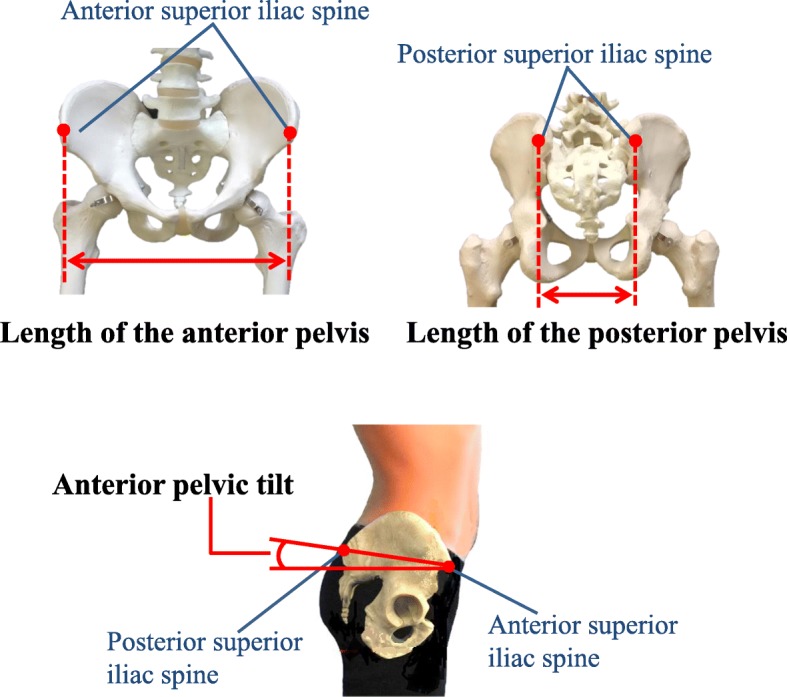
Measurement points for pelvic alignment

Pelvic alignment was measured using a palpation meter (PALM, St. Paul, MN, Performance Attainment Associates, USA). The anterior width of the pelvis in centimeters was measured by placing the PALM caliper tips in contact with the bilateral anterior superior iliac spines (Fig. 2). The posterior width of the pelvis was similarly measured as the distance between the posterior superior iliac spines. The anterior pelvic tilts were measured bilaterally by placing the PALM caliper tips in contact with the ipsilateral anterior and posterior superior iliac spines (Fig. 2). This method is valid, reliable, and cost-effective for calculating any discrepancies between the patients’ landmarks [18]. During the pelvic alignment measurements, the participants took off their shoes and stood with their hands crossed in front of their chests. The left and right anterior pelvic sagittal tilts were measured in degrees.

#### Measured values

The difference in the bilateral pelvic tilts (in degrees) was defined as the pelvic asymmetry value. From the results of the measurements, the changes in pelvic alignment were calculated using the differences in the measurement values from late pregnancy to 1 month after childbirth. In addition, the anterior and posterior pelvic width values were divided by the participant’s height to obtain standardized values for the anterior and posterior pelvic widths.

#### Measurement accuracy

Before the measurements were taken, the measurers were taught the method of proper use of the PALM, which they practiced repeatedly. To verify measurement accuracy, the nine measurers separately measured the pelvic alignment of one woman using the above method. The verification procedure was repeated twice, at a two-week interval. The measurement procedure showed acceptable intra- and inter-rater reliability with intraclass correlation coefficients (ICC 1.1 and ICC 2.1) of 0.998 (95% CI 0.995–0.999) and 0.998 (95%CI 0.992–1.000), respectively, for the anterior pelvic tilt in this study. The ICC 1.1 and ICC 2.1 for the width of pelvis were 0.989 (95%CI:0.971–0.996) and 0.992 (95%CI:0.972–0.999), respectively.

### Statistical analysis

At first, the participants were divided into four groups according to differences in pelvic belt use in late pregnancy and 1 month after childbirth to investigate the differences during and after pregnancy. Women who used the pelvic belt both during pregnancy and after childbirth were categorized as the “Before and After Childbirth” group (BAC group); those who used pelvic belts only during pregnancy were categorized as the “Before Childbirth” group (BC group); those who used pelvic belts only after childbirth were categorized as the “After Childbirth” group (AC group); those who did not use the pelvic belt at all during the maternity periods were categorized as the “Non-Use” group (NU group). One-way analysis of variance (ANOVA) with a post-hoc Tukey test for the interval scale, and a chi-square test for the nominal scale were used to evaluate differences in demographic characteristics such as age, height, weight before pregnancy, weight in each experimental period, number of previous childbirths, mode of delivery, and pelvic alignment (anterior pelvic width, posterior pelvic width and pelvic asymmetry). Next, an initial one-way ANOVA with post-hoc Tukey test was conducted to compare the amount of change in pelvic alignment from late pregnancy to post-childbirth between the groups. After the initial analysis, a multivariate regression analysis was performed to determine the statistically significant differences between the groups after the initial analysis to consider other factors that influenced pelvic alignment. The changes in pelvic alignment that showed statistically significant differences between the groups were specified as the dependent variables. The differences between the BAC and AC groups were specified as the independent variables, and the other factors associated with pelvic alignment (age, BMI in late pregnancy, number of previous childbirths, vaginal delivery and pelvic asymmetry in late pregnancy) [19, 20] were also specified as independent variables. Finally, we determined a set of cutoff points (“grouping cutoff points”) based on the length of time that the pelvic belts were worn. These grouping cutoff points were used to stratify the participants into the study groups. Cutoff points for use or non-use were increased from zero by evaluating the amount of time the pelvic belt was used in a week. Then, a one-way ANOVA with post-hoc Tukey test was conducted to compare the amount of change in pelvic alignment from late pregnancy to after childbirth between each group. Statistical analyses were performed using SPSS version 23.0 (SPSS, Chicago, IL, USA) with a significance threshold set at .05.

## Results

**Fig. 3:**
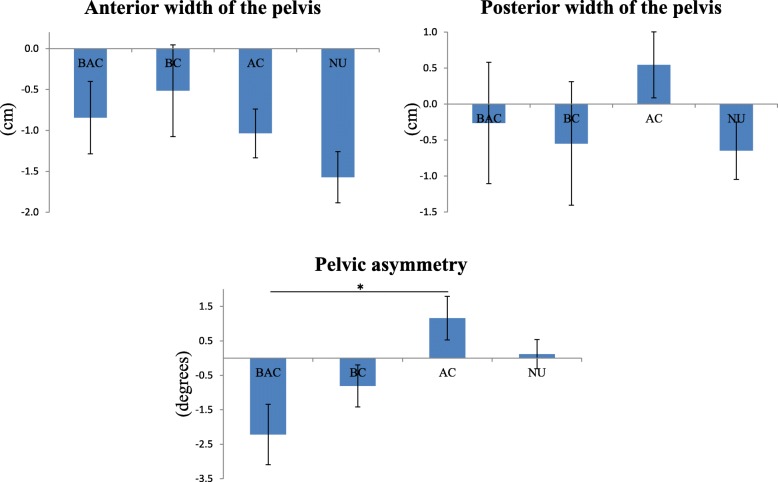
The amount of change in pelvic alignment during pregnancy to after childbirth. BAC: Before and After Childbirth group; BC: Before Childbirth group; AC: After Childbirth group; NU: Non-Use group. *: *p* < 0.05

**Fig. 4:**
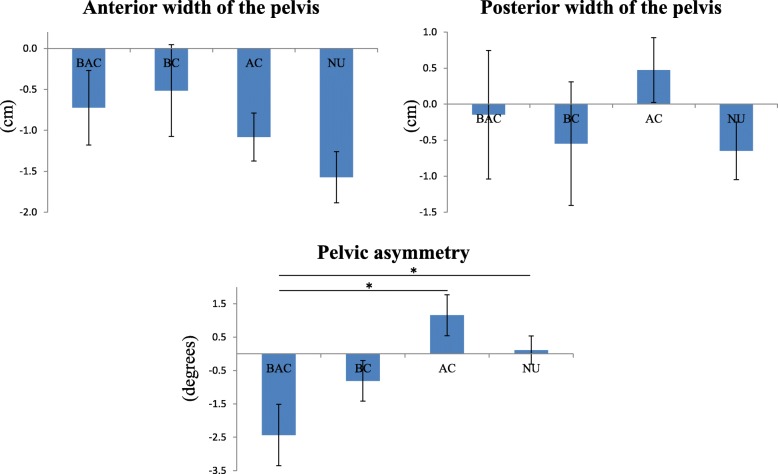
The amount of change in pelvic alignment during pregnancy to after childbirth. Participants who used the belt not less than 7 h weekly were only included in the user group. BAC: Before and After Childbirth group; BC: Before Childbirth group; AC: After Childbirth group; NU: Non-Use group. *: *p* < 0.05

The pelvic belt use rates for all participants of this study were 25.4 and 48.8% in late pregnancy and 1 month after childbirth, respectively. The total sampling rates in the BAC, BC, AC and NU group were 15.9, 10.4, 34.3 and 39.3%, respectively. The demographic data and pelvic alignment for both experimental periods for each group are shown in Table 1. There was no statistically significant difference in the demographic data of each group. In contrast, there were statistically significant differences in some of the pelvic alignments between the differences of pelvic belt use. Regarding the pelvic alignments in the BC group was significantly smaller than that of the AC group at 1 month after childbirth (9.6 ± 2.8 cm, 12.1 ± 4.2 cm, *p* = .032) (Table 1). Similarly, the posterior width of the pelvis divided by the height in AC group was also significantly greater than that of the BC group at 1 month after childbirth (7.6 ± 2.6 cm, 6.1 ± 1.7 cm, *p* = .033). The amount of change in pelvic alignment from during pregnancy to after childbirth is shown in Fig. 3. The decrease in pelvic asymmetry in the BAC group was significantly greater than that of the AC group (− 2.2 ± 0.4 degree, 1.2 ± 0.6 degree, *p* = .003) (Additional file 1). The results of the multiple regression analysis are shown in Table 2. The decrease in pelvic asymmetry from the during pregnancy period to the postpartum period was greater in the BAC group than in the AC group, even after considering other factors which are thought to be related to changes in pelvic alignment during pregnancy (*p* = .024). Then, cutoff points were determined to facilitate stratification of the participants into groups based on the lengths of time the pelvic belts were worn. When the cutoff time was 7 h in a week, the pelvic asymmetry from during pregnancy to postpartum in the BAC group decreased, whereas that for the NU (*p* = .040) and AC (*p* = .002) groups remained unchanged or increased, as shown in Fig. 4 (Additional file 2).

**Table 1:** Demographic characteristics of participants

	All	During pregnancy					All	1 month after childbirth				
	*n* = 201	BAC	BC	AC	NU	*p* value	*n* = 201	BAC	BC	AC	NU	*p* value
		*n* = 32	n = 21	*n* = 69	*n* = 79			*n* = 32	*n* = 21	*n* = 69	*n* = 79	
Age (years)	30.9 ± 4.4	30.4 ± 4.6	31.7 ± 4.5	30.5 ± 3.9	31.2 ± 4.7	0.546	–	–	–	–	–	–
Height (cm)	158.5 ± 5.7	160.4 ± 6.3	158.7 ± 4.8	159.0 ± 5.7	157.4 ± 5.5	0.073	–	–	–	–	–	–
Weight before pregnancy (kg)	52.9 ± 7.6	54.5 ± 8.8	53.5 ± 8.3	52.5 ± 6.9	52.4 ± 7.4	0.542	–	–	–	–	–	–
Weight [during experimental period] (kg)	59.9 ± 7.8	61.7 ± 7.5	60.7 ± 9.7	59.8 ± 7.3	59.1 ± 7.7	0.436	56.1 ± 7.6	57.7 ± 7.8	57.3 ± 9.5	56.0 ± 6.9	55.2 ± 7.5	2.079
Number of previous childbirths (number of people (%))												
0 (primiparity)	83	9 (28.1)	8 (38.1)	33 (47.8)	33 (41.8)	–	–	–	–	–	–	–
1	83	18 (56.3)	6 (28.6)	27 (39.1)	32 (40.5)	–	–	–	–	–	–	–
2	28	5 (15.6)	7 (33.3)	5 (7.2)	11 (13.9)	–	–	–	–	–	–	–
3	5	0 (0)	0 (0)	4 (5.8)	1 (1.3)	–	–	–	–	–	–	–
4	1	0 (0)	0 (0)	0 (0)	1 (1.3)	–	–	–	–	–	–	–
5	1	0 (0)	0 (0)	0 (0)	1 (1.3)	–	–	–	–	–	–	–
Average (number)	0.8 ± 0.9	0.9 ± 0.7	1.0 ± 0.9	0.7 ± 0.8	0.8 ± 1.0	0.630	–	–	–	–	–	–
Anterior pelvic width (cm)	24.9 ± 2.4	25.4 ± 2.6	24.1 ± 1.9	24.8 ± 2.6	25.0 ± 2.4	0.305	23.7 ± 2.6	24.5 ± 3.0	23.6 ± 2.9	23.8 ± 2.5	23.4 ± 2.5	0.211
Anterior pelvic width [divided by height] (cm/m)	15.7 ± 1.5	15.8 ± 1.5	15.2 ± 1.1	15.6 ± 1.5	15.9 ± 1.6	0.251	15.0 ± 1.6	15.3 ± 1.9	14.9 ± 1.7	15.0 ± 1.5	14.9 ± 1.6	0.608
Posterior pelvic width (cm)	11.6 ± 3.5	12.1 ± 3.8	10.2 ± 3.4	11.6 ± 3.3	11.8 ± 3.6	0.224	11.5 ± 3.7	11.9 ± 4.4	**9.6 ± 2.8** ^**†1**^	**12.1 ± 4.2** ^**†1**^	11.2 ± 2.7	**0.040**
Posterior pelvic width [divided by height] (cm/m)	7.3 ± 2.3	7.5 ± 2.3	6.4 ± 2.1	7.3 ± 2.0	7.6 ± 2.5	0.193	7.2 ± 2.3	7.4 ± 2.8	**6.1 ± 1.7** ^**†2**^	**7.6 ± 2.6** ^**†2**^	7.1 ± 1.8	**0.046**
Pelvic asymmetry (°)	3.0 ± 3.0	4.2 ± 4.3	2.9 ± 3.0	2.7 ± 2.7	2.7 ± 2.4	0.080	3.0 ± 3.8	2.0 ± 1.9	2.1 ± 1.8	3.9 ± 4.9	2.8 ± 3.5	0.062
Mode of delivery (number of persons (%))												
Natural childbirth	157	29 (90.6)	16 (76.2)	55 (79.7)	55 (69.6)	0.107	–	–	–	–	–	–
Cesarean section	28	3 (9.4)	3 (14.3)	10 (14.5)	14 (17.7)	0.734	–	–	–	–	–	–
Forceps delivery	8	0 (0)	1 (4.8)	2 (2.9)	5 (6.3)	0.438	–	–	–	–	–	–
Vacuum extraction	5	0 (0)	1 (4.8)	1 (1.4)	3 (3.8)	0.546	–	–	–	–	–	–
Epidural	3	0 (0)	0 (0)	1 (1.4)	2 (2.5)	0.709	–	–	–	–	–	–
Duration of labor [excluding cesarean section] (minute)	472.1 ± 377.8	489.5 ± 374.1	491.9 ± 392.1	501.0 ± 432.0	433.6 ± 326.6	0.779	–	–	–	–	–	–
Birth weight (kg)	3.1 ± 0.4	3.0 ± 0.6	3.0 ± 0.3	3.2 ± 0.4	3.0 ± 0.4	0.096	–	–	–	–	–	–

Grouping by use of pelvic belt; *BAC* Before and After Childbirth group, *BC* Before Childbirth group, *AC* After Childbirth group, *NU* Non-Use group

Values are shown as mean ± standard deviation

The data in bold are statisticaly significant. ^†1^ *p* value for Tukey’s test = 0.032, ^†2^ *p* value for Tukey’s test = 0.033

**Table 2:** Results of the multiple regression analysis

Pelvic asymmetry		
Independent variable	Regression coefficient	95% CI
Age	−0.125	−0.37-0.06
Number of previous childbirths	0.015	−1.07-1.28
Vaginal delivery	−0.040	−3.41-2.11
**Pelvic asymmetry at 30 weeks of pregnancy**	**−0.489**	**−1.10- -0.20**
**BMI at 30 weeks of pregnancy**	**0.184**	**0.02–0.71**
**Use of pelvic belt: BAC or AC**	**0.200**	**0.31–4.20**

The data in bold are statisticaly significant

*CI* confidence interval; *BMI* body mass index, *BAC* Before and After Childbirth group, *AC* After Childbirth group (Grouping by use of pelvic belt)

## Discussion

The current study investigated the influence of pelvic belt use on pelvic alignment during and after pregnancy among pregnant women who did not use a pelvic belt in early pregnancy. The posterior width of the pelvis at 1 month after childbirth in women who used pelvic belts after childbirth was greater than that of who used the pelvic belt before childbirth (Table 1). Regarding the change in pelvic alignment from during pregnancy to after childbirth, the pelvic asymmetry among women who continued to use pelvic belts during and after pregnancy decreased, whereas that for women who only used pelvic belts after childbirth increased (Fig. 3). Moreover, these differences were statistically significant even after adjusting for other factors related to pelvic alignment. In addition, when the cutoff time for pelvic belt use was changed to 7 h per week, the pelvic asymmetry of women who continued to use it during and after pregnancy decreased in contrast of that of women who only used it after childbirth, as well as those who did not use the belt in the perinatal period at all (Fig. 4).

In the current study, women who used a pelvic belt during the recruitment period were excluded to remove the influence of pelvic belt use prior to pregnancy. Nevertheless, about one in four participants in late pregnancy, and half of the participants at 1 month after childbirth used the pelvic belt. Thus, it can be said that there is a substantial demand for pelvic belts among perinatal women. At present, various kinds of pelvic belts are commercially available, whether they are manufactured for perinatal women or not, and an increased awareness might contribute to the high rate of overall use. Moreover, use of pelvic belts have been introduced in some obstetrics and gynecology and healthcare organizations [21, 22], thus, the use of pelvic belts are becoming increasingly widespread. Meanwhile, many women use pelvic belts without really understanding the method of correct usage because of its ease of acquisition and availability in standard markets. Therefore, guides for use and usage assessments among perinatal women are required with the increasing use of the pelvic belt.

Regarding the pelvic alignments in each investigation period, the posterior width of the pelvis, which relates to the opening the pelvis, might be correlated with use of pelvic belt. Specifically, the posterior width of pelvis at 1 month after childbirth among women who used the pelvic belt during pregnancy was less than that among those who used if after childbirth. This might be because the typical shape of pelvic belts for pregnant women has a front-attaching tape, and women usually put the belt on, pulling it from back to front [13, 23]. Many women only fasten the belt in the front of the pelvis, and so the posterior joint might be subject to loosening. Restriction of pelvic joint expansion can have harmful effects as the pregnancy progresses because that process is needed for growth of the fetus and the childbirth process. Although harmful influences such as differences in the mode of delivery were not observed in this study, investigations into whether the pelvic belt is associated with any restriction to the necessary pregnancy-related expansion of the pelvis are needed. In contrast, the difference of change of anterior pelvic width between these groups was not observed significantly. These results might indicate that the pelvic belt use whether during pregnancy or after childbirth is more related to posterior width than anterior width of pelvis after childbirth. Besides, the positive correlation between the change of anterior pelvic width from late pregnancy to after childbirth and birth weight was observed significantly (Additional file 3). In other words, the greater birth weight might be more related to greater values of anterior width of pelvis after childbirth than pelvic belt use.

Regarding the relationships between pelvic belt use and changes in pelvic alignment, there was significantly less pelvic asymmetry among women who continued to use pelvic belts during and after pregnancy, compared to women who used the belt only after childbirth, whose pelvic asymmetry was either increased or unchanged. During pregnancy, a trend toward increasing pelvic asymmetry was observed [24]. In general, the pelvis is naturally closed after childbirth and the condition of the body returns to its nearly original condition [25]. However, the specific changes in pelvic asymmetry after childbirth have not been revealed. Meanwhile, the elasticity of the joints recovers because of decreases in hormone secretion after childbirth [26]. Thus, it can be said that the recovery of pelvic asymmetry is difficult once it increases after childbirth. Since the recovery of pelvic alignment after childbirth is required to prevent some complications after childbirth and for the duration of women’s lives [27], a lack of recovery of pelvic asymmetry could be a serious problem. According to the results of the current study, continuous use of pelvic belts during and after pregnancy might correct increased pelvic asymmetry. One reason why the asymmetry among women who used the belt only after childbirth was significantly greater than that among women who continued to use the belt during and after pregnancy, might be that women who are not accustomed to using the pelvic belt used it incorrectly, which may have contributed to increased asymmetry in pelvic alignment after childbirth [17]. On the other hand, an investigation was also conducted after changing the grouping cutoff point according to the length of pelvic belt use because there were some participants who only used the belt for approximately one or 2 h per week. As shown in the result, the pelvic belt might have almost no effect when the duration of its use is too short. When the participants who used the belt for no less than 7 h were included in the group of users, the pelvic asymmetry among women who did not use the belt, in addition to that among women who used it only after childbirth, was either unchanged or increased, compared to the decrease in pelvic asymmetry among women who continued to use the belt during and after pregnancy. In other words, use of the pelvic belt for less than 7 h per week cannot be proven to affect pelvic alignment. Therefore, continuous use of the belt compared with no use and use only after childbirth might affect changes in pelvic asymmetry in the perinatal period. Hence, continuous use of the pelvic belt during and after pregnancy, especially for longer than a certain length of time in a week, might be associated with modifications of pelvic asymmetry.

There are several limitations in this study, one of which is its observational study design. Additionally, a consistent type and design of the pelvic belt was not specified. For these reasons, definitive effects of pelvic belt use, and also effects of the type of belt on changes in pelvic alignment during and after pregnancy were not evident. Hence, further research that includes an interventional study design is required to support the results of the current study. In addition, we did not evaluate other factors that may have affected pelvic alignment, such as pregnancy-related hormone levels, muscular strength, physical flexibility, or posture of women. Therefore, we would describe this as a pilot study which only suggests effect of pelvic belt use on pelvic alignment during and after pregnancy. Despite these limitations, it can be said that the use of pelvic belts in the perinatal period has some associations with pelvic alignment that may have noteworthy effects on women’s lives.

## Conclusions

In this study, the influence of pelvic belt use on pelvic alignment in the perinatal period was investigated during pregnancy and after childbirth. We found that continuous and prolonged use of pelvic belts during and after pregnancy were related to modifications of pelvic asymmetry in the perinatal period. It is possible that pelvic belt use reduces pelvic widening after childbirth. However, further investigation is needed because there could be some harmful effects related to reduced widening of the pelvis in pregnancy. Therefore, it can be said that guidance in pelvic belt use and also follow-up and evaluation of belt usage during and after pregnancy are important in the management of pelvic mal-alignment among women. Moreover, this is the first study that investigates the effects of pelvic belt use on pelvic alignment based on objective data for pregnant women. The results might be used as a fundamental knowledge for promoting the beneficial use of pelvic belts in perinatal period.

## Additional files

Additional file 1: The amount of change of pelvic alignment from pregnancy to postpartum periods. (XLSX 11 kb)

Additional file 2: The amount of change of pelvic alignment from pregnancy to postpartum periods (The participants who used the belt no less than 7 h were only included to user group). (XLSX 11 kb)

Additional file 3: Results of correlation analysis of change in pelvic alignment and birth weight. (XLSX 11 kb)

## Data Availability

The datasets during and/or analyzed during the current study available from the corresponding author on reasonable request.

## Abbreviations

AC: After childbirth
ANOVA: Analysis of variance
BAC: Before and After Childbirth
BC: Before childbirth
ICC: Intraclass correlation coefficients
NU: Non-use
PALM: Palpation meter
